# Retraction: Inhibition of HMGB1-Induced Angiogenesis by Cilostazol via SIRT1 Activation in Synovial Fibroblasts from Rheumatoid Arthritis

**DOI:** 10.1371/journal.pone.0230889

**Published:** 2020-03-19

**Authors:** Hye Young Kim, So Youn Park, Sung Won Lee, Hye Rin Lee, Won Suk Lee, Byung Yong Rhim, Ki Whan Hong, Chi Dae Kim

Following publication of this article [1], concerns were raised about western blot images in Figs 3D, 4B, and 4E. Specifically,

- In Fig 3D, there is an appearance of vertical discontinuities on either side of lane 4 in the VEGF panel; the β-Actin panel is a duplicate of lanes 1–5 of the β-Actin panel in Fig 4B.

- In Fig 4B and 4E, the β-Actin panels were included as a result of an error in figure assembly. β-Actin is a cytosolic protein, and was not measured in these experiments because NF-κB translocation was measured in nuclear extracts, and results were expressed relative to control untreated cells.

The authors retract this article as it is not possible to fully resolve the concerns regarding Fig 3D because the majority of the underlying data for the article is unavailable.

HYK, SYP, SWL, HRL, WSL, BYR, CDK agreed with the retraction. KWH either could not be reached or did not reply directly.
